# Bioinformatics analysis for the purpose of designing a novel multi-epitope DNA vaccine against *Leishmania major*

**DOI:** 10.1038/s41598-022-22646-7

**Published:** 2022-10-27

**Authors:** Sama Rashidi, Seyed Nooreddin Faraji, Amirreza Javadi Mamaghani, Saeid Hatam, Bahram Kazemi, Peyman Bemani, Seyyed Javad Seyyed Tabaei, Gholamreza Hatam

**Affiliations:** 1 Department of Parasitology and Mycology, School of Medicine, Shiraz University of Medical Sciences, Shiraz, Iran; 2 School of Advanced Medical Sciences and Technologies, Shiraz University of Medical Sciences, Shiraz, Iran; 3 Department of Parasitology and Mycology, School of Medicine, Shahid Beheshti University of Medical Sciences, Tehran, Iran; 4 Science and Technology Park of Fars, ExirBitanic Company, Shiraz, Iran; 5 Cellular and Molecular Biology Research Center Shahid, Beheshti University of Medical Sciences, Tehran, Iran; 6 Department of Immunology, Isfahan University of Medical Sciences, Isfahan, Iran; 7 Basic Sciences in Infectious diseases Research Center, Shiraz University of Medical Sciences, Shiraz, Iran

**Keywords:** Biological techniques, Molecular biology

## Abstract

Leishmaniasis is one of the main infectious diseases worldwide. In the midst of all the different forms of the disease, Cutaneous Leishmania (CL) has the highest incidence in the world. Many trial vaccines have been developed with the purpose of generating long-term cell-mediated immunity to *Leishmania(L) major*. As there is not any multi-epitope DNA vaccine with high efficacy against *L.major*, the aim of this study is to design a new multi-epitope DNA vaccine in order to have effective control upon this infectious disease through the immune bioinformatics. The *L.major* antigens: Gp63, LACK, TSA, LmSTI1and KMP11 were selected to design a multi-epitope DNA vaccine. The initial structure of the DNA vaccine was designed, benefiting from Gen Bank's website information. Epitopes of MHC-I antigens were predicted through the Immune Epitope Database (IEDB), and the selected epitopes were used to make vaccines construct along with linkers. New multi-epitope vaccine including 459 nucleic acids designed, and inserted between *BamH1* and *HindIII* restriction sites of pCDNA3.1 mammalian expression vector. 12 epitopes among the chosen antigens were selected by two servers (IEDB and ANTIGEN). They had high stability and high antigenic power. Physicochemical features of vaccine measured by ProtParam server, and this structure was thermostable and hydrophilic. it’s a suitable model to study on the animal and human phases. The designed vaccine is expected to be an effective candidate through development of (CL) vaccines. However, the effectiveness of this vaccine should also evaluate in vivo model.

## Introduction

Leishmaniasis is a disease caused by a parasite of the genus *Leishmania* (L), which varies from a self-healing form such as cutaneous leishmaniasis (CL) to lethal visceral leishmaniasis (VL)^1^. Treatment of leishmaniasis currently involves the use of a number of drugs such as pentamonial antimony, amphotericin B, miltefosine, paromomycin, which in most cases have side effects^2^. Vaccination is the most effective way to eradicate this infectious disease^3^. Structure of various types of vaccines, such as killed parasite vaccine, subunit vaccines and DNA vaccines have been studied by scientists^4^.There are very few Leishmania vaccines that have entered the clinical phase. Therefore, we need more studies to find a good way for the treatment^5^. In order to produce an effective vaccine, it is important to know the host’s immune system. To limit the CL infection, the strong produced cytokines, particularly IFN-γ, play important roles. Stimulation of T helpers, the (Th1) and (Th2) cytokines, is seen in patients with the CL infection. Among them, the Th1 has been reported to be more effective. Furthermore, the macrophages show significant capabilities in defense process. They are triggered by cytokines derived from T-cells, which are vital for controlling or aggravating the disease. Macrophages are a kind of antigen-presenting cell (APC) that distinguishes antigens of the *Leishmania*^6^.As a results, there is a critical requirement for development of a vaccine, that can bring more efficient immunity than the earlier vaccines^7^. Along with the various types of studied vaccines, the use of DNA vaccines has demonstrated promising outcomes. However, the use of these antigens in the form of proteins goes hand in hand by some problems, including high production price, short half-life, poor immunogenicity, low stimulation of cellular immunity, and cross-allergic reactions^8^. The first-generation vaccine of the CL, consists of live attenuated and killed fragmented parasites. Which is just a class of human prophylactic VL vaccine, that has entered the phase III of clinical trials until now. however, this vaccine was not successful to achieve satisfactory results^9^. The second-generation vaccines produced by recombinant *Leishmania* antigens. Among a range of approaches the LEISH-F3, a multicomponent vaccine formulated with GLA-SE adjuvant, illustrated promising results in phase I as a strong immune response inducer in healthy individuals^10^. More recently, a third-generation DNA vaccine that employs simian adenovirus, expressing a new synthetic gene that encode *Leishmania* antigens, have termed as ChAd63-KH. It has indicated potentials to be a protective and immunogenic therapeutic vaccine for the human VL, and the post kala-azar dermal leishmaniasis (PKDL) in phase I trial^11^. Regardless of the current progress in vaccine development, the desirable aim has not yet been attained, that is the development of a safe, effective, durable and low-cost prophylactic vaccine for human leishmaniasis^12^. The use of epitopes or epitope-containing peptides is advantageous, since epitopes are assessed for immune recognition and epitope specific response^13^. Since peptides themselves remain poorly immunogenic, the approaches that are attracting increasing interest are based on the development of peptide-based formulations in combination with potent adjuvant components (peptide, lipids, virus particles, nanoparticles etc.)^14^. Peptide vaccine studies, which just a few years ago were becoming more and more marginalized, are again rising as a guaranteed finding for designing more logical vaccines^15^. While there is an increase in the number of new peptide-base vaccines, a major challenge is how to avoid inactivation or degradation by the immune system, and enhancing the immunogenicity of those peptides. Thus, it is essential to design vaccines using diverse approaches besides the use of other adjuvants, that can help to improve the antigen immunogenicity^16^.

## Methods

### Sequence and structure retrieval

We evaluated *L. major* antigens and the selected antigens were Glycoprotein 63 (Gp63), Leishmania-activated C-kinas (LACK), Thiol-Specific Antioxidant Antigen (TSA), Leishmania major stress-inducible protein1 (LmSTI1), Kinetoplastid membrane protein 11 (KMP11), to design a new multi-epitope DNA vaccine. The protein sequences were obtained by using the corresponding Gen Bank server NCBI, (http://www.ncbi.nib.gov/genbank), which is the protein database of the National Center for Biotechnology Information. The sequence IDs of GP63, KMP11, LACK, LmSTI1 and TSA were ACL01096.2, XP003722616.1, AAA97577.1, AAB37318.1 and AAC31146.1 respectively. All sequences from diverse isolates downloaded from the Gene Bank in the FASTA format. The sequences of all antigenic proteins were checked manually and those unrelated sequences removed.

### Antigenicity analysis

The antigenicity of the selected proteins was evaluated using two online servers, the Vaxijen and the AntigenPro at http://www.ddg-pharmfac.net/vaxijen/VaxiJen/VaxiJen.html and http://scratch.proteomics.ics.uci.edu/, respectively. The VaxiJen predicts the antigenicity of a protein by using a new alignment-independent method and the accuracy of this method is almost 88%. In the case of the Protein’s organism of source, a particular model selected that has a specific threshold. The VaxiJen considers a parasite protein as an antigen, when its score is more than the defined threshold which is 0.5 for the parasite data set. ANTIGENpro is a sequence-based and alignment-free predictor that has not designed for a specific pathogen group. It predicts the antigenicity of protein based on eight features sets and five machine-learning algorithms by using protein antigenicity microarray data. The accuracy of this method is near 76%.

### NCBI BLASTp screening

To prevent probable cross-reactivity with human proteins, the predicted CD8 + T cell epitopes screened against human proteome by NCBI BLASTp at https://blast.ncbi.nlm.nih.gov/Blast.cgi?PAGE=Proteins.

### T-cell epitope prediction

The specific epitopes for major histo compatibility complex (MHC-I) were predicted by using immune epitope database (IEDB) server at http://tools.iedb.org/mhci/.For this purpose, H2-Dd alleles were selected as the mouse MHC-I molecules.

### Construction of leish21 vaccine

T-cell epitopes were linked together via gsgg, ggs, gsgs, ggssgg and ggsg linkers. The prediction of the secondary structure was performed by using an online server, the PSIPRED, available at (http://bioinf.cs.ucl.ac.uk/psipred). Antigenicity Prediction of the leish21, the designed vaccine, utilized by immune epitope database server (IEDB).

### Physicochemical features of vaccine

Physicochemical characteristics of the Leish21 vaccine like the molecular weight (Mw), isoelectric point (pI), instability index and the half-life, were measured by the ProtParam server available at (http://web.expasy.org/protparam).

### Tertiary structure prediction and evaluation

The 3D-structure of the whole protein vaccine modeled using the QUARK serve at https://zhanggroup.org. The QUARK models are built from small fragments by replica-exchange Monte Carlo simulation, through an atomic-level knowledge-based force field, and is suitable for proteins that do not have homologous templates. The multi-epitope peptide vaccine was designed and the HindIII and BamHI restriction sites were used to the N and C terminal of the DNA sequence of the peptide structure, respectively.

## Results

### T-cell epitope prediction

The T-cell’s epitopes prediction identified using the online server (IEDB) based on MHCI binding. Among all the predicted epitopes, only 12 epitopes with higher scores selected. We used three epitopes from each of the Gp63, KMP11 and Lack, one epitope from the LmSTI1and two epitopes from the TSA (Table1).

**Table 1:** Predicted epitopes for KMP11, Gp63, Lack, LmSTI1 and TSA. They selected based on the (IEDB) server.

Allele	Protein	Start position	End position	Sequence	score	antigenicity	hydrophobicity	hydrophilicity	immunogenicity	toxicity
H2_Dd	KMP11	1	9	KLDRLDEEF	0.010	− 1.1514	− 0.43	− 1.33	0.21236	Non-Toxin
H2_Dd		2	10	KMQEQNAKF	0.011	0.7709	− 0.41	− 1.70	− 0.14872	Non-Toxin
H2_Dd		2	10	HFKQKFAEL	0.010	0.5538	− 0.21	− 0.76	− 0.19484	Non-Toxin
H2_Dd	Gp63	2	10	GLPPYWQYF	0.068	0.4954	0.10	− 0.44	0.11452	Non-Toxin
H2_Dd		2	10	VTHEMAHAL	0.043	− 0.0175	0.03	0.32	0.09023	Non-Toxin
H2_Dd		2	10	YGCDTLEYL	0.036	− 0.3372	− 0.02	− 0.07	0.02724	Non-Toxin
H2_Dd	Lack	1	9	IGFSPNRFW	0.092	0.9564	− 0.03	− 0.18	0.33743	Non-Toxin
H2_Dd		3	10	FSPNRFWM	0.087	0.7431	− 0.11	− 0.48	0.307	Non-Toxin
H2_Dd		1	8	IGFSPNRF	0.077	1.4489	− 0.08	− 0.09	0.27139	Non-Toxin
H2_Dd	LmSTI1	1	9	KDPNNTLYI	0.098	1.2882	− 0.23	− 1.08	0.00861	Non-Toxin
H2_Dd	TSA	1	9	RSVEEVLRL	0.087	− 0.3078	− 0.32	− 0.09	− 0.03328	Non-Toxin
H2_Dd		1	9	VCPTEVIAF	0.073	− 0.3357	0.20	1.58	− 0.10168	Non-Toxin

### Construction of the Leish21 multi-epitope DNA vaccine

**Figure 1:**
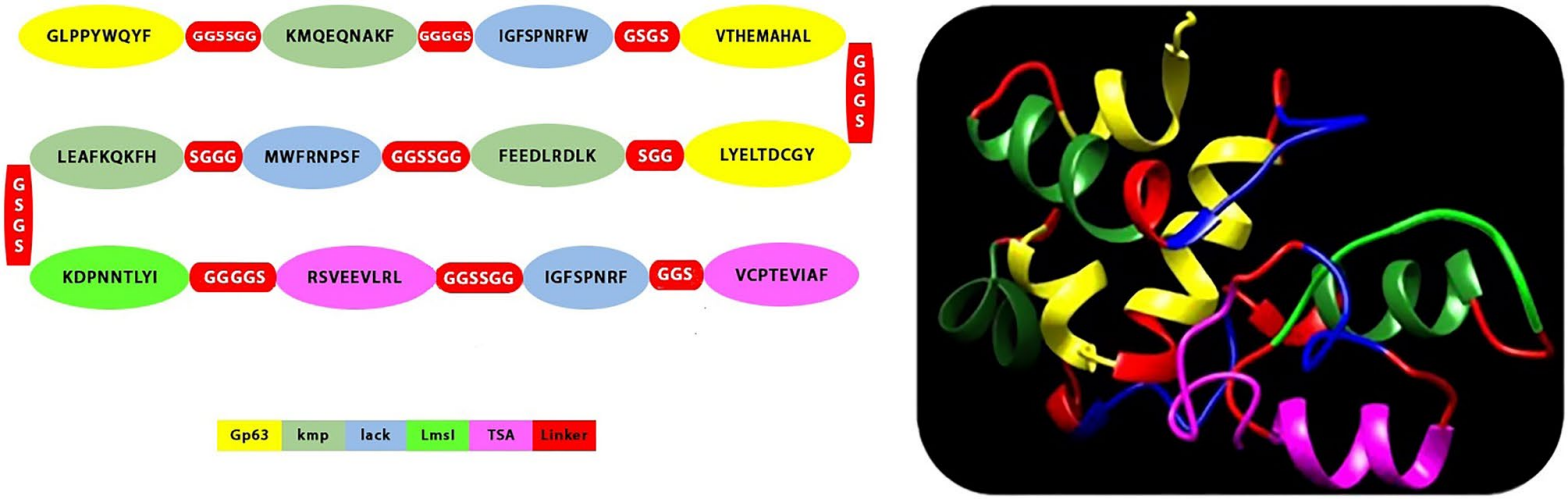
The final epitopes for DNA vaccine designed by several servers and use of linkers between them.

**Figure 2:**
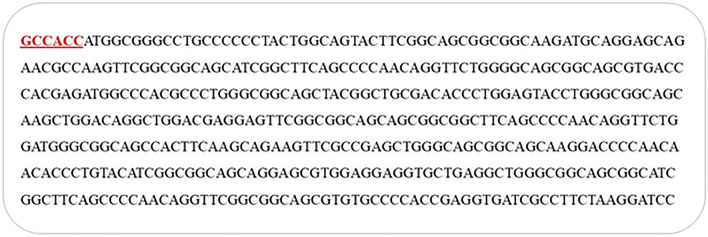
Reverse translation of multi epitope amino acid sequence into nucleotide sequence by using the J-CAT; Location of Kozak piece (red section).

These epitopes with high binding affinity were fused together. The new multi-epitope DNA vaccine, including 459 nucleic acids synthesized. Kozak (GCCCC) is the essential site for initiation of replication. We used Alanine amino acid to improve stability of this structure (Fig. 1, 2). The Leish21 inserted between *BamH1* and *HindIII* restriction sites of the pCDNA3.1 (https://www.snapgene.com/resources/plasmidfiles/?set=mammalian_expression_vectors&plasmid=pcDNA3.1_Hygro(%2B)&format=png ) of mammalian expression vector.

### Antigenicity Evaluation of the leish 21 vaccine

To predict the antigenicity of this structure, VaxiJen v.2 and ANTEGENpro servers were used. The prediction by the VaxiJen was 0.58 and 0.57 by the ANTIGENpro (Table 2). According to the obtained results, the multi-epitope DNA vaccine can activate the immune response.

**Table 2:** Evaluation of antigenicity with two servers (Vaxijen and Antigen) show that leish 21 can activate the immune response.

Vaxijen		Antigen pro
Probability of antigenicity	Probable antigen	Probable antigen
Score	0.58	0.57

### Secondary structure of leish21

**Figure 3:**
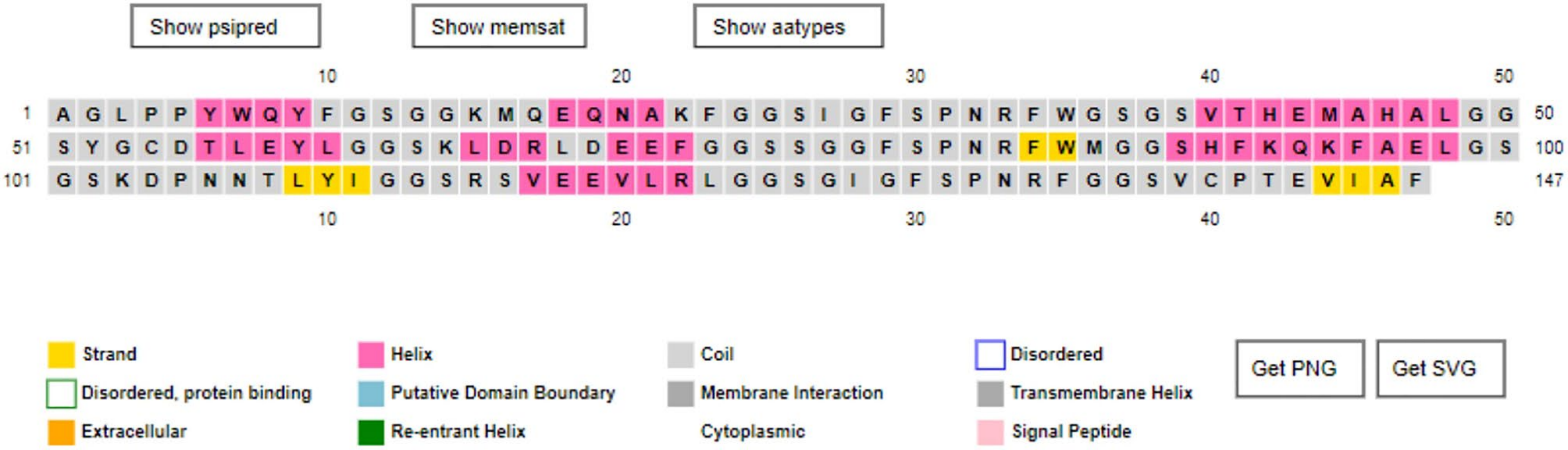
Predicted secondary structure via PSIPRED online service.

Prediction of the secondary structure was performed by PSIPRED server with 29.93% alpha-helix, 5.44% beta strand and 64.63% random-coil which is shown in (Fig. 3).

### Physicochemical parameters of evaluation

**Figure 4:**
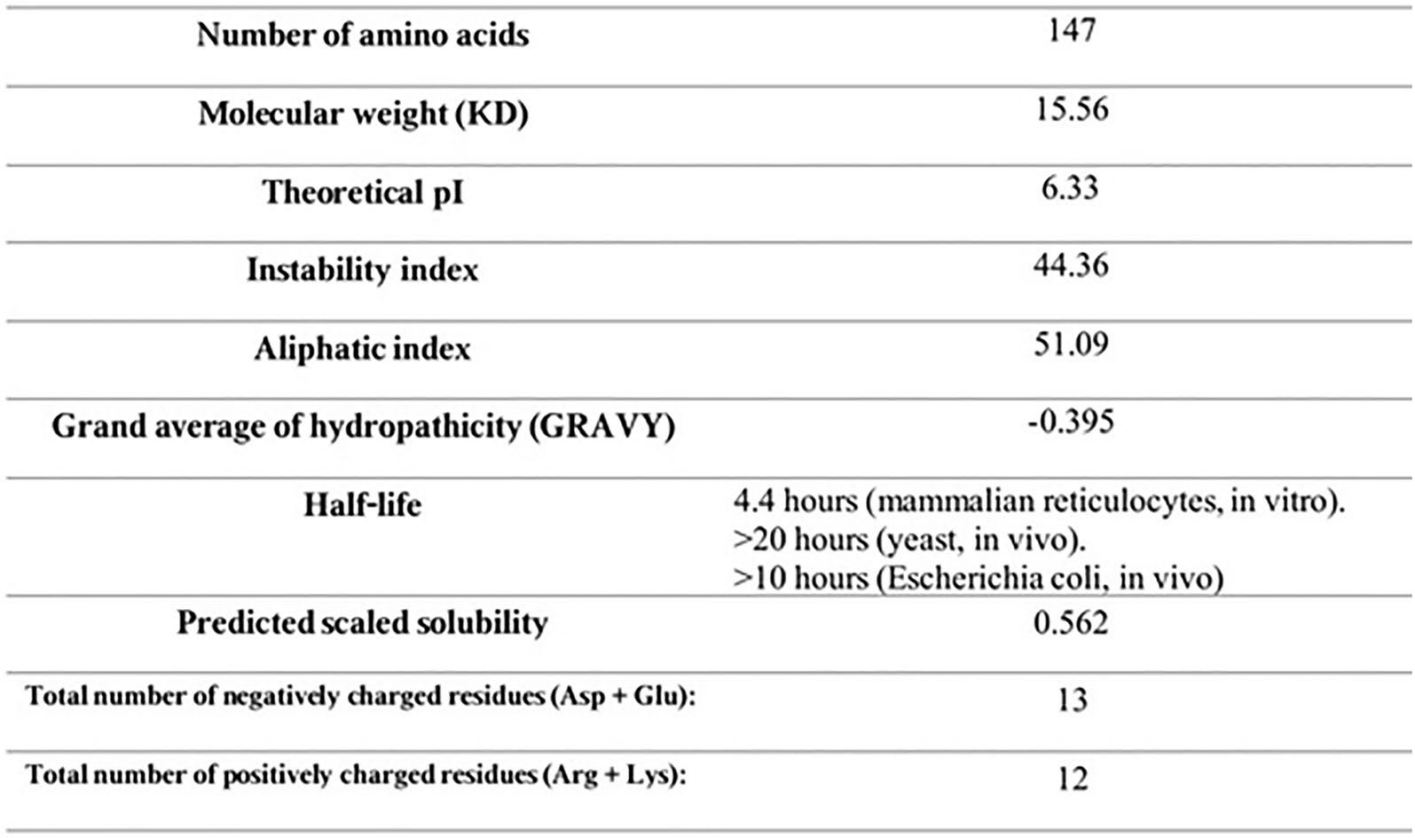
Physico-chemicalparameters evaluation by ProtParam server.

Referring to the ProtParam server, the multi-epitope DNA vaccine consists of: 147 amino acids with the molecular weight (MW) of 15.56 KD, theoretical Isoelectric point (PI) of 6.33 and 2124 number of atoms. Additionally, the predictable half-life in mammalian reticulocytes in vitro was 4.4 h. Instability index, aliphatic index and grand average of hydropathicity (GRAVY) were 44.36, 51.09 and 0.395 respectively (Fig. 4). Therefore, this structure was thermostable and hydrophilic.

### Tertiary structure prediction, refinement, and validation

**Figure 5:**
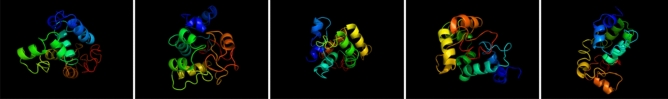
3D structures prediction of leish21 by using QUARK server.

**Figure 6:**
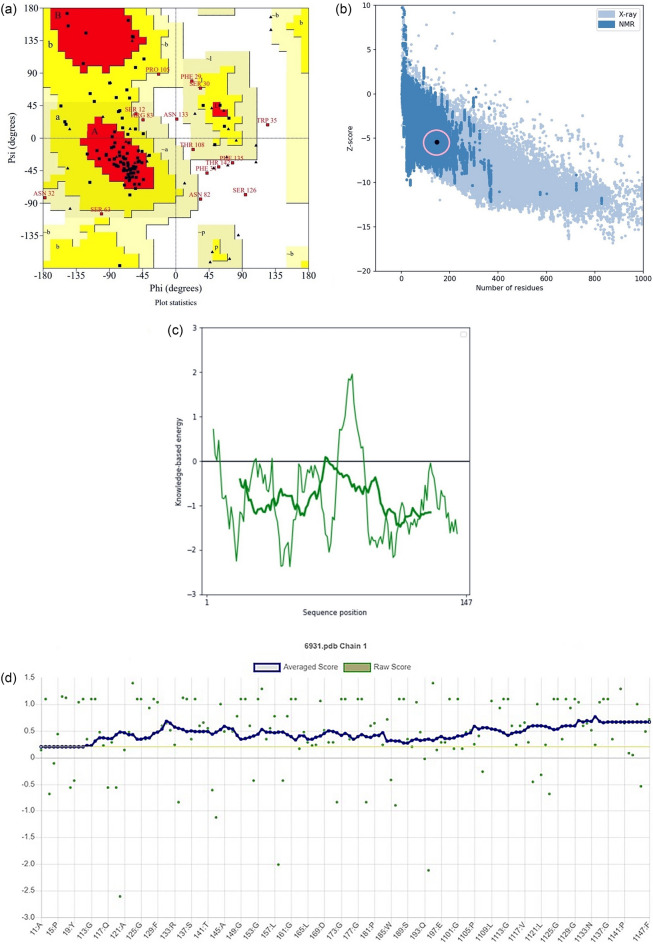
(**a**) Validation of leish 21protein structures by a Ramachandran plot; (**b** and **c**) ProSA-web z-score plot and local model quality for 3D structure of leish21protein structures, respectivel; (**d**) Verify3D method to evaluate a 3D model of final multi-epitope.

The 3D structures of the predicted epitopes modeled with the QUARK server. From the top 5 models predicted by this server, the one with the highest C-score (− 1.93) was selected for further works (Fig. 5). The results of the Ramachandran analysis demonstrated that 65.7% were in favored in the allowed regions. The number of residues in allowed regions were (21.3%), generously allowed regions were (6.5%) (Fig. 6a). The model quality which was performed by the ProSA-web server revealed that the z-score was −5.42. The leish21’s protein structures were also located within the normal range for the three-dimensional structures by the NMR and X-ray method (Fig. 6b). Moreover, the plot of residue scores indicated the local model quality by plotting energies for the leish21 protein structures (Fig. 6c). As most of the residues had a negative score and were below the threshold, so the leish21protein structures had the lowest problematic or erroneous parts. According to Verify3D, least 80% of the amino acids have scored ≥ 0.2 in the 3D/1D profile using, indicating to have a valid structure. (Fig. 6d)^17^.

## Discussion

While leishmaniasis is one of the main health issues in many countries, development of protective vaccines against it, is becoming a big concern. Peptide vaccines are potentially a new technology to struggle with the Leishmania infection. Bioinformatics procedures for the analysis of proteins have become an essential instrument for vaccine researches^13,17,18^. The prediction of epitopes is a significant facility for designing new vaccines^4^.Nowadays use of immune-informatics software, have led to save a great deal of time to design vaccine constructs. Numerous studies have carried out for epitope predictions on microorganisms such as viruses, parasites and bacteria^17,19^. Selecting several epitopes from protein antigens has several advantages over designing vaccines using full–length proteins. One of the advantages is that expressing full length of some proteins to obtain soluble protein form is difficult due to their large molecular weight, so it is better to use epitopes rather than whole protein. Moreover, some regions in the outer part of antigenic proteins have similar sequences with the host proteins and cause autoimmunity and cross- reaction with auto-antigens. Certain regions of the full-length protein also may have immunosuppressive properties. For instance, by inducing regulatory T cells, the parasite’s immune escape happens, and even leads to delay in the elimination and persistence of the parasite. By size controlling of the protein that can induce a stronger immune response, epitopes from more antigens can be used. This makes it possible to consider a large number of combinations of B cell and T cell epitopes, which can trigger a powerful humoral immune response with the help of CD4 Th cells^20^. So far, there is not a definite treatment report for the disease caused by *L. major*. The greatest strategy is vaccine development that can trigger the immune system with respect to intracellular parasite fragments and activation of TC and TH^21^. In addition to diminishing the intensity of reinfection, immunity against *Leishmania* through activating CD4+ T cells and CD8+ T cells play an important role in limiting the infection^22^. Multiple epitope-based vaccines have potential advantages when compared with vaccines using single or combinations of recombinant proteins. Vaccine design can be performed by selecting the most antigenic CD4+ and CD8+ T-cell epitopes from immunogenic Leishmania proteins, and therefore the avoided epitopes were those which presented low immunogenicity or detrimental immune dominance in the whole proteins^23^. Additionally, positives of multiple epitope-based vaccines consist of safety, low manufacturing price, easy quality control process, (and the chance of rationally engineering of the epitopes, to increase their immunogenicity^24^. In the present study, a new multiple epitope DNA vaccine has designed for the *L. major* to be considered as an important choice in reducing this infectious disease. Previous studies pointed out the efficacy of vaccines based on *LACK* combined with other components, including IL-12 and IL-18 in a murine model. The Leish21 should be tested for the immunogenicity and the protective effects in rodent models and maybe for protection of human in the future. One of the selected genes is LACK, which has various functions, such as signal transduction, DNA replication, RNA synthesis and cell cycle control in eukaryotes, and it is expressed in promastigotes and amastigotes. Vaccination by LACK facilitates the initiation of the protective response against CL, due to shifting from Th2 cytokine responses to Th1. The reason for this effective response is that the IFN-γ production is inducted by interleukin 12. Hence, it has been as an appropriate method for improvement of vaccines for leishmaniosis^25^. The ‘GGGS’ constructed linkers causes increasing humoral and cellular immune responses in BALB/c mice. Former studies indicated that in addition to the epitopes, the linker between epitopes is crucial in the efficiency of vaccines, with G-rich linkers being particularly competent for multi-epitope vaccine constructure. G-rich linkers have the capacity to improve sequence flexibility without affecting the function of proteins they attach to. In addition, glycine and serine are high flexibility, and have less side branches compared to other amino-acids. The presence of alcoholic factor, (OH) in serin, can be dissolved in the hypotonic solutions. Immunogenicity of the DNA vaccines on Leishmaniasis evaluated in BALB/c mice, and the immune response provoked in animals^26^. Several physiological characteristics have compared based on previous studies and this study. Aliphatic index of a protein is defined as the relative volume occupied by aliphatic side chains (alanine, valine, isoleucine and leucine). It may be considered as a positive factor for increasing the thermal stability of globular proteins. In a study conducted by Khatoon and Daneshi, it showed that the stability of protein against heat was much higher than other studies. In our study it has shown that the molecular weight of the design construct is lower than the protein size of other studies because in this study, fewer and more specific epitopes were used, which caused the molecular weight to be low^27^(Table 3)

**Table 3:** Comparsion between this study and other studies physicochemical properties of protein structures (instability index، Molecular weight، Isoelectric point (pI) and Aliphatic index).

physiological characteristics	Half life	Instability index(II)	Molecular weight	Isoelectric point(pI)	Aliphatic index
Our study	4.4 h (Mammalian reticulocytes, in vitro) > 20 h (yeast in vitro) > 10 h (Escherichia coli, in vivo)	44.36	15.56 KD	6.33	51.09
Vakili, Bahareh, et al	20 h (Mammalian reticulocytes, in vitro) 30 min (yeast in vitro) > 10 h (Escherichia coli, in vivo)	39.38	45.4 KD	5.96	66.41
Khatoon, Nazia,et	30 h (Mammalian reticulocytes, in vitro) > 20 h (yeast in vitro) > 10 h (Escherichia coli, in vivo)	26.3	54.62KD	9.04	83.48
Salehi-Sangani G,et	3.5 h (Mammalian reticulocytes, in vitro) 10 min (yeast in vitro) > 10 h (Escherichia coli, in vivo)	32.92	38.7 KD	9.44	68.61
Rabienia M,et	30 h (Mammalian reticulocytes, in vitro) > 20 h (yeast in vitro) > 10 h (Escherichia coli, in vivo)	31.23	27.170 KD	4.26	52.06
Daneshi R,et	30 h (Mammalian reticulocytes, in vitro) > 20 h (yeast in vitro) > 10 h (Escherichia coli, in vivo)	29.68	60.08KD	5.17	76.17

It should be noted that comparison between former studies and ours about the assessment of the designed vaccine shows that the molecular weight of the Leish21 is lower than others, and it makes an important advantage for us. Because the possibility of loop production is much lower than other vaccines. In addition, selected epitopes in this study have the highest score in the IEDB. Also, different antigens were used to design this vaccine, which this method is better than the way single-antigen vaccines are designed because it increases the efficacy of a vaccine. Aliphatic index of our vaccine has a lower quantity in comparison with the Khatoon et. al.’s, and this is because of the different molecular weight of their structures. About the vaccine-efficacy comparison with other designed vaccines, it should say that as different vaccines have positives and negatives, while they are not tested in clinical phase a proper comparison is not possible. An ideal vaccine against Leishmaniasis should have a number of potentialities, like: being immune, immunization against different types with limited number of injections for a longer duration, lower production cost, and possibility to be used both for prevent and treatment^28^. To design an effective vaccine for cutaneous leishmaniasis, bioinformatics methods, online servers, and numerous software were utilized for prediction of T cell epitops. Thus, more investigations by using in silico and in vivo patterns must be done in the future studies for assessing potency of the polytopes as a final vaccine choice.

## Conclusion

In this study, immune bioinformatics tools were employed to develop a new multi-epitopes vaccine which obtained from five immunogenic proteins: KMP11, GP63, LACK, LmSTI1 and TSA of the *L. major*. Leish21 need to further be tested on murine models and requiring more studies on this vaccine.

## Data Availability

The authors confirm that the data supporting the findings of this study are available within the article and its supplementary material.
